# Filling Teeth

**Published:** 1857-12

**Authors:** J. Taft


### FILLING TEETH.

BY J. TAFT.

### FORMING CAVITIES.

The next step in the operation is the formation of the cavity. By this the cavity is so formed that it will retain the filling when properly introduced. In very few cases is the cavity of proper form when the decay is removed. Much time, patience, and labor is required of the operator, for the proper accomplishment of this part of the work, and much endurance on the part of the patient.

Excavation is often required to give a regular form to the cavity. In the excavation of cavities, there are several points worthy of consideration. The great object, however, is to give to the cavity such a form as will most certainly retain the filling in place. The cutting for the formation of the cavity should be accomplished with the least possible loss of healthy dentine; this is a point upon which a good judgment should be exercised. The strength of the walls of the cavity, and the ability of the parts to withstand the pressure, both in the introduction and consolidation of the filling and in the act of mastication, should be well noted. It may be regarded as a rule from which there should scarcely ever be a departure, that the enamel should never be encroached upon in excavating to give form to a cavity. When there is but a lining of dentine at any given point on the enamel, after the decay is removed, it should remain for the preservation of the enamel; it should not be cut through either by pits or grooves, much less should any considerable portion be removed.

There are cases occasionally in which the dentine is wholly decayed, and its removal lays bare the enamel; when this occurs, the enamel should be retained as perfect as possible, and no attempt made to form pits or grooves in it. The reason for this is found in the friability of the enamel.

It may be regarded as an axiom, that where it is necessary to cut the healthy dentine to give proper form to a cavity, it should be done at that part of the cavity where the tooth will suffer least from the loss. The precise point and amount of cutting will be determined by the form and size of the cavity, and the amount of solid dentine remaining after the decay is removed.

In small cavities, where there is sufficient material to work upon, the object is to give the cavity a regular form, and make the retaining points where it is most convenient.

In large cavities, where one side of the tooth is weak, places must be selected for making the retaining points, that will not affect the weak point. Frequently, in approximal decays of the anterior teeth, the labial and palatal walls are friable, and would be easily broken; much cutting upon such walls would not be admissible. Again, the decay often extends toward the point of the tooth, down to the union of the labial and palatal plates of the enamel; in cases of this kind, all that can be done at this point is to remove the decay; and fracture will sometimes occur, even in accomplishing this.

In some cases, as in the crown cavities of the molars, the cavity will be nearly or quite of proper form when it is perfectly opened up, and the decay all removed. This is the case when the decay is confined to a simple perforation of the dentine, without any lateral extensions.

In approximal cavities, there is always more or less excavation of the solid dentine required, to give the cavity proper form.

There is no definite rule for the form of cavities, that will be applicable in all cases. The form will be modified by the tooth, the position of the decay upon the tooth, and the extent and ramifications of the decay. It is given as a rule by some that the depth of a cavity should be equal to its least diameter. This is a direction, however, of no general application, for many cavities will be much deeper than the greatest diameter, as in crown cavities of the molars, and the reverse will often occur, as in labial cavities of the superior incisors, and in approximal cavities of the molars, when it would be impossible to make anything like an approximation to this rule, without exposing the nerve, and even cutting through the nerve chamber.

A general direction, and one which we think good, and applicable in many cases, especially in crown cavities of the molars, and almost any of the deep perforations by decay, is to make the walls of the cavity as nearly parallel as possible with one another. This rule is applicable in almost all small cavities.

In medium or large-sized cavities, it is admissible to leave them slightly larger at the bottom than at the orifice, if circumstances require; a large cavity of this form can be perfectly filled, when a small one could not, from the fact that there is more room to manipulate with the instrument, in introducing and consolidating the filling.

Cavities that are larger within than at the orifice, should have their walls perfectly plain, smooth surfaces, free from transverse grooves or depressions, so that the gold may be perfectly adapted to them.

It is sometimes necessary to leave a cavity slightly larger at the orifice than at the bottom. This may be done by a converging inclination of the wall of one or more sides of the cavity. When there is an inward inclination of the wall at one side of the cavity, the general form may be such as to retain a filling perfectly, for there may be two opposite sides parallel, or even diverging; in that case, the axis of the cavity will not be in the direction of the center of the crowns.

Two opposite sides may converge and the others diverge, and a filling be retained firmly. When two contiguous sides have the same converging inclination, making the orifice larger than within, if the walls are smooth, plain surfaces, a filling will not be retained; but retaining points may be made by forming transverse grooves, or pits upon them, and by this means the filling be firmly retained. As a general rule, it will be necessary, when the orifice is larger than the cavity within, to make grooves or pits on the walls. For these the proper points should be selected.

If the cavity is large, and the walls near the orifice, thin, and liable to be broken, the situation of the grooves or under cutting should be farther within the cavity than if the walls are firm out to the edge. Sometimes it is best to make little pits at the bottom of such cavities for retaining points.

In cases where it is necessary to make an under-cutting, one or two little transverse grooves upon one side will be sufficient, and in no case on more than two sides, leaving the others perfectly plain surfaces.

In the formation of retaining points in difficult cavities, there is considerable diversity of practice—under-cutting and grooving have been employed very commonly. Another method has been very frequently adopted during the last three or four years, viz: drilling little holes or pits into the dentine at the most favorable points, these taking different directions.

This kind of retaining points is much better calculated to answer the purpose, in filling with crystal gold, or adhesive foil by Dr. Arthur's method, than with the ordinary foil after the old methods.

When different inclinations are given to these perforations, and then perfectly filled with adhesive gold of any kind, the filling would certainly be retained in place.

For making these perforations, an English broach and a small square-edged drill are quite sufficient.

This class of retaining points are seldom or never required in crown cavities of the molars; but in approximal cavities they are frequently employed with great advantage.

In forming them, great care should be exercised, lest the nerve chamber is approached by the instrument. In almost all cases, the proper point for forming them is at the bottom of the cervical wall of the cavity.

Another particular to which attention should be given is the border of the orifice. It should always be an object to secure an *even*, *smooth*, and *strong* border to the orifice of the cavity. It is impossible to make a good finish with a rough, uneven border; the filling is also more exposed to injury by mastication. The integrity of a smooth, plain surface is perfectly retained under influences that would break up and destroy an uneven one.

It is also very desirable to have a *firm* margin; to obtain this, it is often necessary to cut away more than would otherwise be desirable. A smooth, firm border should not be sacrificed for the form, and especially so in regard to the posterior teeth.

It is very objectionable to some persons to have the perfect form of the front teeth marred or changed; but it should be remembered that even a front tooth, one third cut away, and so filled as to retain it permanently, is far more valuable than an artificial tooth.

Another particular that should always be regarded, is to avoid all acute angles. These are seldom or never found in approximal cavities of the molars and bicuspids; occasionally they are found in approximal cavities of the cuspids, and frequently in approximal cavities of the incisors, particularly at that part of the cavity next to the cutting edge of the tooth. And such angles are very often found in crown cavities of the molars, where there is an extension of the decay along one or more of the fissures of the crown.

It is difficult--almost impossible—to fill perfectly a sharp angle, and hence the necessity of obliterating them when they occur. This may be done either with a small delicate cutting instrument, or a small burr drill. It is an operation requiring great care and delicate manipulation, at least so far as the anterior teeth are concerned. When a sharp angle occurs in the approximal cavities of the front teeth, it is usually near the cutting edge of the tooth, just at the union of the labial and palatal plates of enamel. A small chisel shaped instrument is very good for cutting out such angles. For cutting out such points in fissures of crown cavities of molars, where the decay extends backwards, the straight, chisel-shaped instrument is just adapted; but when there is an anterior extension, the instrument should be curved to almost a right angle, and forced down by pressure of the thumb of the left hand.

Some good operators recommend a slight reaming at the orifice of all cavities, where it can be accomplished. The object for this is two-fold: to remove the sharp angle at the orifice of the cavity, as it is liable to be roughened in putting in the filling; and to give a better border to the filling.

In making this bevel, the burr, if one is used, should be but little larger than the orifice of the cavity. The cutting should be but slight,—just sufficient to remove the sharp corners; much cutting here would give too thin and yielding edge to the filling.

### DRYING CAVITIES.

After a cavity is properly formed it should be thorougly cleaned and dried. Every particle of detached bone or foreign substance should be removed; during the excavation everything should be kept out, every fragment removed as soon as it is detached; but generally there is something of the kind to remove after the cavity is formed. This may be done probably better with syringe than by any other method; this, however, used in connexion with a moist lock of cotton on a probe, will serve to remove every extraneous material that may be in the cavity.

Any extraneous substance remaining in the cavity, prevents the perfect adaptation of the gold to the part, and consequently as perfect attachment as would otherwise be obtained. After washing out thoroughly, wipe out with successive locks of dry cotton, until all the moisture is removed. The ordinary cotton will not accomplish this very perfectly. By washing cotton in sulph. ether, it is much improved for this purpose. The ether removes a peculiar oily substance from it, and thus increases its ability to absorb moisture. Ether or chloroform will either answer the purpose; or boiling the cotton in water in which there is a small portion of carb. soda, or some such alkali, for a short time, will accomplish the same thing.

Tissue paper is used, and, by some, preferred to cotton. Blotting paper is also used, and preferred by some, to anything else. Prepared flax has likewise been used. Either of these is no better than cotton well prepared. The respect in which the paper is any better than common cotton, is, that it has the same treatment in effect as the prepared cotton.

With none of these things can a cavity be made absolutely dry. It is not necessary to have absolute dryness to make a good filling, yet a more perfect filling can be made where that condition is obtained.

Gold takes a better hold upon perfectly dry, than upon a moist surface. This is quite apparent in the following experiment. Place two or three blocks of gold in any ordinary cavity, wiped as dry as possible, and press them firmly in place, consolidating them as much as would be done in filling, then test the attachment by removal; then reduce the same cavity to absolute dryness, take the same amount of gold, in the same form, and place it in the cavity, and consolidate as before, at the same point in the cavity; then test its attachment, by removal. It will be much more firmly attached than the first portion.

But it is objected that this is useless, inasmuch as the natural moisture of the tooth is removed, by producing absolute dryness, and that this will soon return, and then the cavity will be no dryer than it could have been made with good cotton or paper. Admitting this, perfect dryness will remain long enough for the introduction of the filling. And if this is facilitated by absolute dryness, and a better adaptation and attachment obtained, then it *is* desirable to obtain that condition.

It has already been remarked that perfect dryness can not be obtained with cotton or paper. Warming these on a hot metallic plate, after being wrapped on the instrument, will very much increase their efficiency in removing moisture.

Asbestos wrapped on a small bulb-pointed instrument, and heated, is very good for drying out cavities; with it perfect dryness can be obtained, as it can be re-heated and applied as often as necessary.

To prepare this instrument, select a proper sized bulb-pointed instrument; a worn out burr drill answers well, and fold over it fibers of asbestos, passing them a little way beyond the bulb on the shaft of the instrument, where it is bound firmly on with fine platinum wire, and the instrument is ready for use. Another method of obtaining perfect dryness—one which is very certain in its result—is to throw a jet of warm air into the cavity. This is accomplished by a little instrument, simple in structure and easily used. It consists of a small blow-pipe with a cylinder an inch long, and half an inch in diameter; this is placed down within two inches of the point of the instrument. This cylinder is either made of very heavy metal, or filled with wire or something that will retain heat; upon the other end is attached a stiff india-rubber ball with an opening one and a half lines in diameter. By placing the thumb upon this opening, and making compression, a jet of air is forced through the point of the pipe, and the cylinder being previously heated, the temperature of the jet will correspond with that of the cylinder, and the velocity with which it is forced through the instrument. This jet thrown into a cavity, which has been made as dry as possible by wiping, soon makes a very apparent change, the walls of the cavity becoming whiter than before. This we consider the most perfect condition in respect to dryness, that can be obtained.

